# Impact of high atmospheric carbon dioxide on the biotic stress response of the model cereal species *Brachypodium distachyon*

**DOI:** 10.3389/fpls.2023.1237054

**Published:** 2023-08-16

**Authors:** Lug Trémulot, Catherine Macadré, Joséphine Gal, Marie Garmier, Alexandra Launay-Avon, Christine Paysant-Le Roux, Pascal Ratet, Graham Noctor, Marie Dufresne

**Affiliations:** ^1^ Université Paris-Saclay, CNRS, INRAE, Université Evry, Institute of Plant Sciences Paris-Saclay (IPS2), Gif sur Yvette, France; ^2^ Université Paris Cité, CNRS, INRAE, Institute of Plant Sciences Paris-Saclay (IPS2), Gif sur Yvette, France; ^3^ Institut Universitaire de France (IUF), Paris, France

**Keywords:** climate change, carbon dioxide, biotic stress, plant immunity, *Brachypodium distachyon*, *Magnaporthe oryzae*, salicylic acid, transcriptome

## Abstract

Losses due to disease and climate change are among the most important issues currently facing crop production. It is therefore important to establish the impact of climate change, and particularly of high carbon dioxide (hCO_2_), on plant immunity in cereals, which provide 60% of human calories. The aim of this study was to determine if hCO_2_ impacts *Brachypodium distachyon* immunity, a model plant for temperate cereals. Plants were grown in air (430 ppm CO_2_) and at two high CO_2_ conditions, one that is relevant to projections within the coming century (1000 ppm) and a concentration sufficient to saturate photosynthesis (3000 ppm). The following measurements were performed: phenotyping and growth, salicylic acid contents, pathogen resistance tests, and RNAseq analysis of the transcriptome. Improved shoot development was observed at both 1000 and 3000 ppm. A transcriptomic analysis pointed to an increase in primary metabolism capacity under hCO_2_. Alongside this effect, up-regulation of genes associated with secondary metabolism was also observed. This effect was especially evident for the terpenoid and phenylpropanoid pathways, and was accompanied by enhanced expression of immunity-related genes and accumulation of salicylic acid. Pathogen tests using the fungus *Magnaporthe oryzae* revealed that hCO_2_ had a complex effect, with enhanced susceptibility to infection but no increase in fungal development. The study reveals that immunity in *B. distachyon* is modulated by growth at hCO_2_ and allows identification of pathways that might play a role in this effect.

## 1 Introduction

In the context of an increasing population, crop production is a key issue for food security. Crops are facing pathogen attacks with the impact of diseases representing around 17-30% of current losses (Savary et al., 2019). The impact of pathogens reflects characteristics of three components depicted in the disease triangle: the host, the pathogen, and environmental factors (Francl, 2001). The environmental component is likely to be profoundly modified by ongoing climate changes. One of the most prominent environmental changes is the increase of atmospheric carbon dioxide (CO_2_). According to the IPCC’s worst projection, CO_2_ concentration could rise to more than 1000 ppm by the end of the century (2100) (Solomon et al., 2009; Fuss et al., 2014).

Since CO_2_ is the basic substrate for photosynthesis, and is currently sub-saturating for C_3_ plants, an increase in atmospheric CO_2_ concentration impacts plant physiology. Notably, C_3_ plants show reduced photorespiration and increased photosynthesis as the oxygenase activity of RuBisCO decreases relative to carboxylase activity (von Caemmerer, 2020). However, many studies have shown that this increase in photosynthesis flux does not reach the theoretical rate and that the increase in growth and development is not as high as expected. A similar effect is observed for crop yields, with results of Free-Air CO_2_ Enrichment (FACE) experiments showing a yield increase that is ~50% less than predicted (Woodward, 2002; Long et al., 2006; Ainsworth and Long, 2021). However, even if increased CO_2_ concentration leads to acclimation, it has been shown to modify plant physiology, raising the question of its impact on plant immunity in C_3_ plants.

The well-known trade-off between growth and immunity emphasises the relevance of studying plant immunity in conditions that promote enhanced growth such as hCO_2_ (Huot et al., 2014). In addition, changes in primary metabolites such as amino acids have been shown to be important for establishing compatible interactions and acting on components of plant immunity (Liu et al., 2010; Stuttmann et al., 2011). Increased primary metabolism may enhance secondary metabolism due to increased concentrations of substrates (Noctor and Mhamdi, 2017). There is therefore a clear potential metabolic link between hCO_2_ and immunity, since numerous metabolites synthesised by secondary metabolism pathways are involved in the plant response to biotic stress (Balmer et al., 2013). Previous studies have reported an up-regulation of pathways related to specialised defence metabolites at hCO_2_ concentrations (Noctor and Mhamdi, 2017; Vicente et al., 2019). This effect is particularly apparent for phenylpropanoids and terpenoids, two major classes of secondary metabolites that include phytoalexins and other compounds involved in defence. Further, modified metabolism may affect redox homeostasis, possibly leading to changes in the concentration of reactive oxygen species, which can be important signalling components (Foyer and Noctor, 2020).

Recent studies have shown that hCO_2_ can modulate plant immunity in a positive or negative manner, depending on the plant species and the pest or pathogen (Kazan, 2018). Previous studies have mainly focused on dicotyledons. For example, in Arabidopsis, plant susceptibility to pathogens can be decreased by hCO_2_ and evidence has been presented that this is linked to altered redox signalling, stomatal closure, and accumulation of resistance-related compounds, notably salicylic acid (SA) (Mhamdi and Noctor, 2016; Noctor and Mhamdi, 2017; Williams et al., 2018; Foyer and Noctor, 2020). For this, a key question is whether models developed from work on dicotyledonous plants can be applied to monocotyledons. It is crucial to determine whether hCO_2_ impacts plant immunity in the same generic manner in all flowering plants or if there are specificities linked with different phylogenetic groups. Although many immunity components are conserved, some dissimilarities have been reported (Han, 2019). Studying monocots is relevant from the food security point of view since many economically important crops belong to this group. Indeed, cereals represent around 60% of all calories obtained by humans, either directly or indirectly *via* meat consumption (Cassman et al., 2003).

*Brachypodium distachyon* (Bd) has emerged as a very useful model for cereals, which still present several obstacles to easy study. It has a close phylogenetic relationship and a high degree of synteny with cereal crops, and advantages include its easy cultivation in laboratory conditions and the availability of bioinformatic and genetic resources (Opanowicz et al., 2008; Scholthof et al., 2018). *Brachypodium* also shows a compatible interaction with various important cereal pathogens (Fitzgerald et al., 2015), such as *Magnaporthe oryzae*, the causal agent of rice blast (Parker et al., 2008). This hemibiotrophic fungi pathogen can be inoculated at the vegetative stage on leaves and accesses plant tissues through mechanical penetration. This last characteristic enables immunity to be studied independently of complicating factors such as the potential impact of hCO_2_ on stomatal aperture and density (Xu et al., 2016; Zhang et al., 2018).

The aim of this study was to assess the impact of hCO_2_ on *Brachypodium* through a combined approach using phenotyping, transcriptomics, resistance tests, and SA assays. We used two hCO_2_ concentrations, one that is relevant to short-term predictions of atmospheric composition (1000 ppm) and a second that is sufficient to largely suppress photorespiration and saturate photosynthesis (3000 ppm). Together, the data reveal intriguing effects of different CO_2_ concentrations on gene expression and point to a complex impact of hCO_2_ on biotic stress pathways and resistance in this monocotyledonous plant.

## 2 Materials and methods

### 2.1 Plant material and growing conditions

*Brachypodium distachyon* ecotype Bd21-3 was cultivated in a growth chamber under a 20 h light period at 24 ± 1 °C under fluorescent light (200 ± 20 μmol.m^−2^.s^−1^ at the soil level). The humidity level was 65 ± 1 %. Before sowing, seeds were surface sterilised by incubation in a 0.6 % sodium hypochlorite solution for 10 min with gentle shaking followed by three rinses in sterile distilled water. Sterilised seeds were subsequently incubated for 5-7 days at 4 °C in the dark. Plants were grown routinely on a 3:1 mixture of compost (Tref terreau P1, Jiffy France SARL, Trevoux, France) and standard perlite (Nestaan, Tholen, the Netherlands), soaked with an aqueous solution containing the ProPlant^®^ fungicide (Fargro, Arundel, UK) and the Steirnernema-system larvicide (Biobest, Westerlo, Belgique). Plants were usually watered in two- to four-day intervals using a standard nutritional solution (Plant-prod 14-12-32, 280 g/L and Fertiligo 4.35 mL/L). Growth under hCO_2_ conditions was achieved by growing plants in a Snijders growth chamber (Microclima MC1000E) with a CO_2_ concentration of 430 ppm, 1000 ± 100 ppm or 3000 ± 200 ppm.

### 2.2 Plant phenotyping

At the vegetative (21 days after sowing) and mid-anthesis (33 days after sowing) stages, the shoot phenotype of plants grown at the three CO_2_ concentrations was assessed. At 21 days, shoot length, tiller number, leaf number and shoot fresh weight were measured. At mid-anthesis, shoot length, shoot fresh weight, spikes number and number of spikelets per spike were measured.

### 2.3 *Magnaporthe oryzae* growth, infection assays, symptom scoring and biomass quantification

*M. oryzae* P1.2 strain was maintained at 26 °C on rice flour agar medium (Saleh et al., 2012). Ten days-old plates were scraped, and the collected material was resuspended in 3 mL of sterile Milli-Q^®^ water. Conidia were collected by filtration onto sterile Miracloth (Calbiochem^®^), and the recovered spore suspension was adjusted to a final concentration of 10^5^ spores.mL^-1^ in 0.01% Tween 20. Whole shoots of 21 days-old plants were sprayed with the fungal spore suspension until run-off. Inoculated plants were transferred to the infection growth chamber (8 h light; 20 °C light and 18 °C dark; humidity 60-80 %; ambient air). During the first 48 hours, a transparent lid was placed over the plants to ensure saturating hygrometry. At 5 days post inoculation (dpi) the third youngest leaf of each plant was sampled. At least five leaves were sampled per replicate and condition, flattened, and scanned for image analysis. Leaf and lesion areas were quantified using Fiji (Schindelin et al., 2012). Given the increase of total leaf surface under hCO_2_, for each infected leaf, the percentage of diseased leaf area was corrected using the following formula:


$$\%\;diseased\;leaf\;area=100\times \;\frac{whole\;lesions\;area}{mean\;control\;leaf\;surface}$$

where *control* corresponds to leaves from plants grown in ambient air.

For each of the three biological replicates in each condition, four leaves were pooled for fungal biomass quantification and frozen in liquid nitrogen. Genomic DNA (gDNA) extraction was performed as described below. DNA purification was performed using the Genomic DNA Clean & Concentrator Kit^®^-25, following the supplier’s instructions, including Note number 5 (ZymoResearch^®^). qPCR was performed on 20 ng total gDNA using 8 pmol of each primer specific for *M. oryzae* 28S rDNA and for *B. distachyon UBC18* gene (**Table S1**) and 10 µL of SYBRGreen Master Mix in a final volume of 20 µL. Reactions were performed in a Light Cycler LC480 real-time PCR system (Roche Diagnostics). All qPCRs were carried out on biological triplicates, each in technical duplicate. The final C_t_ values were means of three values (biological triplicates), each corresponding to the mean of technical duplicates. The Light Cycler^®^ LC480 real-time PCR system set with default parameters automatically determined the Ct for each reaction. The Light Cycler^®^ LC480 Software was used to determine the quantity of fungal or plant gDNA by referring to Ct values obtained for standard curves of pure gDNA from either *M. oryzae* or *B. distachyon* and the corresponding specific primer pair (see above and **Table S1**). The specificity of the qPCR was determined by melt-curve analysis of the amplified products using the standard method installed in the system.

### 2.4 Transcriptomic analysis

The whole shoots of 21-day-old Bd21-3 grown at the different CO_2_ concentrations were sampled. RNA extraction was performed as described above. 4 µg of total RNA was purified using RNA Clean & Concentrator™-5 following the supplier’s instructions (ZymoResearch^®^). The RNA-seq transcriptomic analysis was performed by the POPS platform (IPS2, INRAE, France) as follows. The RNA integrity was verified by microarray Agilent (Agilent RNA 6000 Nano Kit). Following the supplier’s instructions, the libraries were constructed using the TruSeq Stranded mRNA kit (Illumina^®^, Californie, USA). Messenger RNAs were purified and fragmented before the first and second-strand RT steps. Then, sequencing adaptor tags were ligated to the cDNA. After a new purification step, the cDNAs were amplified by PCR and validated on Agilent DNA HS microarray. The libraries were then multiplex-sequenced with the Next-Seq500 kit, with an average reading length of 150 bases. A first bioinformatic treatment was performed, leading to the identification of differentially expressed genes (DEGs) according to a Wald test (Benjamini-Hochberg adjusted p-value<0.05). A table with gene accessions, annotations (Annotation *Brachypodium distachyon* v3.1 34 310 genes), log_2_ fold change (log2FC) and counts according to genes and conditions was provided.

### 2.5 Data accession number

The RNA-seq dataset of the Illumina reads has been deposited in the NCBI Gene Expression Omnibus under accession number GSE229886.

### 2.6 Bioinformatic analysis of the transcriptomic data

A co-expression analysis was performed on R by clustering, followed by an enrichment analysis on the clusters for KEGG pathways (Kanehisa and Goto, 2000) and Gene Ontology terms (The Gene Ontology Consortium, 2019). This analysis was done for the DEGs in at least one condition and on Z-score normalised counts. The clustering analysis was done by comparing several clustering methods (K-means, Hierarchical clustering, Fuzzy clustering, Generalised linear model; **Table S2**) and cluster numbers (from 5 to 30) determined by the Silhouette coefficient (Rousseeuw, 1987) and Davies-Bouldin index (Davies and Bouldin, 1979). AIC and BIC were used for the optimal method to confirm the cluster number (Akaike, 1974; Schwarz, 1978). For each cluster, the enrichments are determined using the clusterProfiler package (Wu et al., 2021). The background used corresponds to the genes sequenced, and the p-value cut-off is 0.05 for Fisher’s exact test.

A targeted analysis was performed on DEGs, focusing on gene families and gene members associated with signalling and metabolic pathways. Orthologs for the DEGs considered, in *O. sativa* and *A. thaliana*, are assigned, if possible, based on sequence similarity using Phytozome (https://phytozome-next.jgi.doe.gov/; “Brachypodium distachyon v3.1” and “Arabidopsis thaliana Araport11”) and EnsemblPlants (https://plants.ensembl.org/Oryza_sativa/).

### 2.7 Total SA quantification by HPLC

For total SA quantification, 5-6 leaves (the 3^rd^ youngest) of plants grown 21 days at the three CO_2_ concentrations were pooled per repetition and condition and then frozen in liquid nitrogen. SA quantification was performed by HPLC-fluorescence, as previously described (Simon et al., 2010).

## 3 Results

### 3.1 *B. distachyon* phenotype at high CO_2_

**Figure 1:**
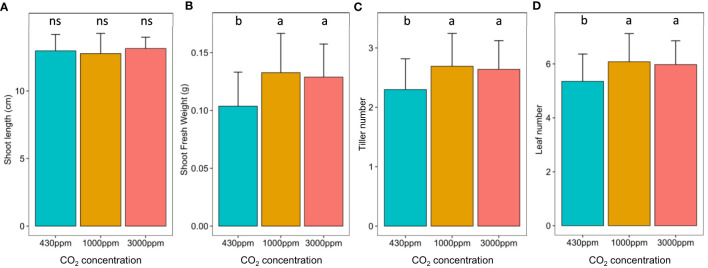
Impact of hCO_2_ on *B distachyon* development. *B distachyon* shoot length **(A)**, shoot fresh weight **(B)**, tiller number **(C)** and leaf number **(D)** at the vegetative stage (21 days after sowing). Different letters mean a significant difference according to a Kruskal-Wallis and *post-hoc* Dunn test with a Benjamini-Hochberg correction (adj-pvalue < 0.05). Error bars correspond to the standard deviation.

At the vegetative stage, hCO_2_ did not impact shoot length (**Figure 1A**) but did result in significant increases in shoot weight, tiller and leaf number, with both hCO_2_ concentrations producing similar effects (**Figures 1B–D**). Effects of hCO_2_ were also observed at the reproductive stage (**Figure S1**). The shoot length was increased at hCO_2,_ most notably at 1000 ppm, while the shoot weight increased at 1000 ppm but decreased at 3000 ppm. Spike number followed the same trends as shoot weight. On the other hand, the number of spikelets per spike increased significantly at 3000 ppm. Hence, while developmental differences were observed under hCO_2_, these were more pronounced at the reproductive stage, when in addition to impacting parameters relative to the control, the two hCO_2_ concentrations also differed from each other.

### 3.2 Impact of growth at hCO_2_ on *B. distachyon* susceptibility to *M. oryzae*

To assess if growth at hCO_2_ impacts Bd susceptibility at the vegetative stage, leaves were inoculated with the leaf fungal pathogen *M. oryzae* and the infection was assessed at 5 dpi. We chose this hemibiotrophic pathogen because of its economic impact and because it displays a biotrophic phase followed by a necrotrophic state, requiring the plant to call on different responses to resist these two phases (Pieterse et al., 2012).

**Figure 2:**
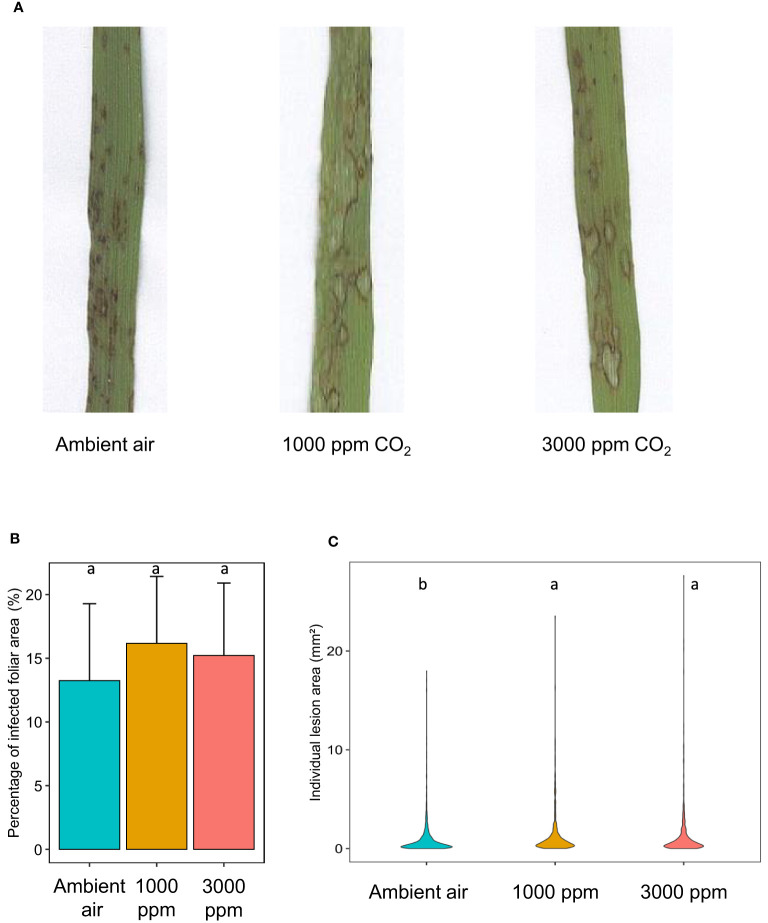
*B distachyon* leaves exhibit increased *M. oryzae*-induced lesion area at hCO_2_. Representative lesions **(A)**, percentage of infected foliar area (%; **(B)** and individual lesion area (mm²; **C)** at 120 hours post-infection (hpi) of *B distachyon* plants grown at ambient air, 1000 or 3000 ppm CO_2_ during 21 days before fungal inoculation. Different letters mean a significant difference according to an ANOVA and *post hoc* Tukey’s HSD test **(B)** or a Kruskal-Wallis and *post hoc* Dunn test with Benjamini-Hochberg correction **(C)** (adj-pvalue < 0.05).

Growth at hCO_2_ did not impact the overall percentage of diseased leaf surfaces at 5 dpi (**Figure 2B**) even after correction taking into account the increase in total leaf surface at hCO_2_ (**Figure S2**). To directly determine fungal development, *M. oryzae* biomass was quantified in diseased leaves. The mean fungal biomass was higher, but not significantly, at hCO_2_ compared to ambient air (**Figures S3 A, B**).

However, individual lesions were larger after growth at hCO_2_ than observed in the control condition (**Figure 2C**). This was visually apparent at 5 dpi (**Figure 2A**) and confirmed by the quantified increase in individual lesion area, which was around 50 % higher at hCO_2_ than in ambient air (**Figure 2C**).

### 3.3 Salicylic acid content in *B. distachyon* at high CO_2_

**Figure 3:**
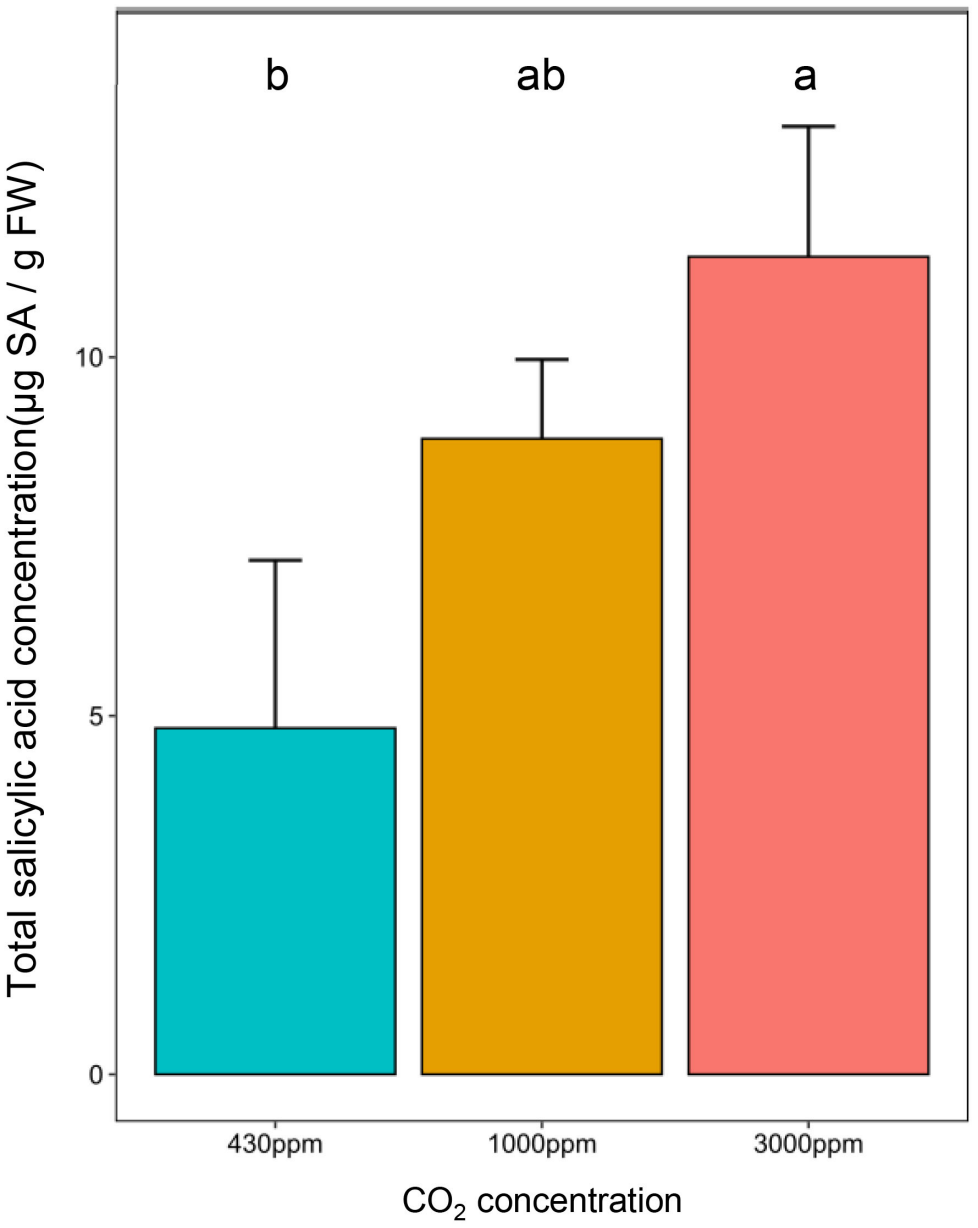
High CO_2_ induces total SA accumulation in 21 days-old *B distachyon* leaves. Total salicylic acid (SA) concentration in µg of total SA per gram of fresh weight (g FW) in the second youngest leaf according to CO_2_ concentrations. Different letters correspond to a significant difference according to a Kruskal-Wallis and Dunn *post-hoc* test with a Benjamini-Hochberg correction (adj-pvalue < 0.05). Error bars correspond to standard deviation.

The modulation of plant susceptibility by growth at hCO_2_, and other reports in which this condition has been reported to increase SA, led us to investigate whether the amount of this key immunity-related phytohormone was modified. Indeed, total SA content was more than 2-fold higher at 3000 ppm CO_2_ than in the control condition (430 ppm). The phytohormone concentration at 1000 ppm was intermediate between those quantified at the other two concentrations, although not significantly different (**Figure 3**).

### 3.4 *B. distachyon* transcriptome at high CO_2_

**Figure 4:**
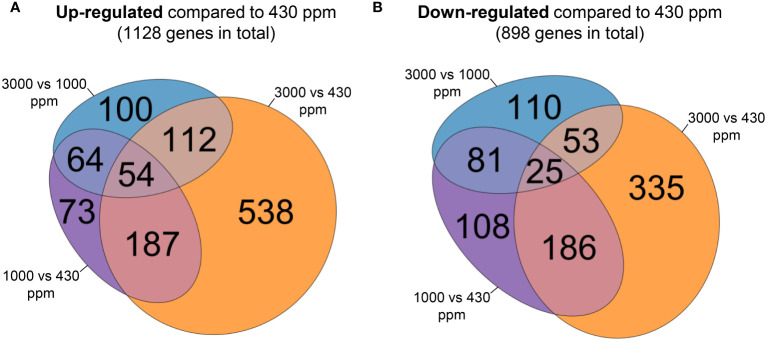
Venn diagrams comparing differentially expressed genes (DEGs) between the 430, 1000 and 3000 ppm CO_2_ growing conditions. Each diagram corresponds to DEGs up-regulated **(A)** or down-regulated **(B)** at 1000 or 3000 ppm CO_2_ compared to 430 ppm CO_2_ using raw counts. For each diagram, the area of each set is proportional to the number of DEGs comprised in it.

To explore how hCO_2_ modifies the Bd transcriptome, and to assess which pathways are impacted by hCO_2_, we performed RNA-Seq analysis to compare the shoot transcriptomes of plants grown in ambient air or at elevated CO_2_ concentrations (1000 and 3000 ppm) for 21 days. Analysis allowed the identification of 1726 DEGs. Of these, 300, corresponding to 17 % (300 among 1726), showed opposite trends at hCO_2_: down-regulated at 1000 ppm and up-regulated at 3000 ppm compared to 430 ppm CO_2_, or the other way around. These DEGs are thus present in both diagrams (**Figure 4**). Among the 1726 DEGs, the expression of 48 % (828 genes = 1128 – 300/1726) was found to show a general enhancement by increases in growth CO_2_. The percentage of DEGs that were generally repressed by higher CO_2_ was 35 % (598 genes = 898 – 300/1726).

A clustering of the 1726 DEGs based on Z-score normalised counts expression patterns was performed to obtain a global overview. According to an analysis based on the Davies-Bouldin index and Silhouette coefficients, the best performing method was found to be K-means using an optimal cluster number of nine (**Figures S3, S4**). The nine clusters generated, each comprising between 54 and 390 DEGs, show different expression profiles according to the three CO_2_ concentrations (**Figure S5**; **Table S5**). While two included genes progressively repressed by hCO_2_ (2 and 4, comprising 381 genes), three clusters grouped genes that were enhanced by hCO_2_ (6, 8, and 9, 948 in total). The remaining clusters (1, 3, 5, and 7, 397) revealed a more complex response, in which the direction of the effect was dependent on CO_2_ concentration (**Figure S5**).

**Figure 5:**
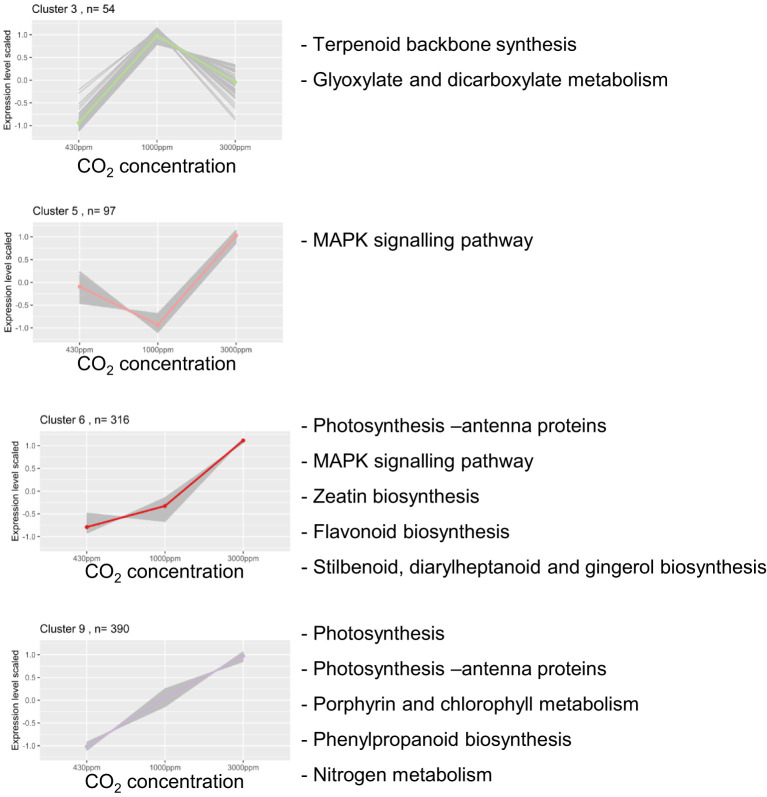
Four gene clusters are enriched for KEGG pathway terms. Scaled counts for clusters showing KEGG pathway enrichment (p-value < 0.05). For each cluster, the coloured line corresponds to the mean of DEGs expression. “n=“ corresponds to the number of DEGs in each cluster. On each graph from left to right is depicted the scaled counts value for 430, 1000 and 3000 ppm CO_2_.

The clusters were then further analysed for, first, gene ontology and, second, KEGG pathway enrichment (**Table S3**). The first showed significantly enriched terms for all clusters except 2 and 7, while the second highlighted four clusters showing significant enrichments (clusters 3, 5, 6 and 9) and is shown in **Figure 5**. These clusters included the two largest ones showing enhanced gene expression by hCO_2_ (6, 9; **Figure 5**).

We further mined the DEGs based on (1) the enrichment described above; (2) our analysis of the effect of hCO_2_ on fungal susceptibility and SA contents in Bd; and (3) the potential involvement of redox homeostasis in responses to hCO_2_. The text below outlines the major features of this analysis.

#### 3.4.1 Primary metabolism and reactive oxygen species

Several photosynthesis-related genes were up-regulated in the Bd transcriptome under hCO_2_ conditions. This result is highlighted by the KEGG enrichment related to this pathway in clusters 6 and 9. These enrichments correspond to genes encoding proteins of the photosynthesis electron transport chain: components of photosystem I, photosystem II, ATPase, and chlorophyll and light-harvesting protein complexes. On the other hand, two genes coding for RuBisCO subunits (*Bradi1g39206*, *Bradi3g26391*) were found in cluster 3, characterised by an increased gene expression at 1000 ppm but a decrease at 3000 ppm (**Figure 5**). Alongside RuBisCO, components of carbon metabolism (TCA cycle, glyoxylate and dicarboxylate metabolism) were also enriched in cluster 3. Strikingly, it should be remarked that there was a GO Biological process term enrichment for “trehalose biosynthetic process” in cluster 4, which shows repression at hCO_2_ (**Table S3**). This enrichment corresponds to five of the nine genes annotated as “trehalose phosphate synthase” in Bd.

Genes involved in nutrient assimilation were increased under hCO_2,_ as highlighted by the “Nitrogen metabolism” term enrichment for cluster 9 (**Figure 5**; cluster 9). The genes encoding nitrate and nitrite reductase (*Bradi3g37940*, *Bradi3g57680* and *Bradi3g57990*, respectively), two enzymes allowing nitrate (N) assimilation, were gradually up-regulated under increasing CO_2_ concentrations.

#### 3.4.2 Secondary metabolism

**Figure 6:**
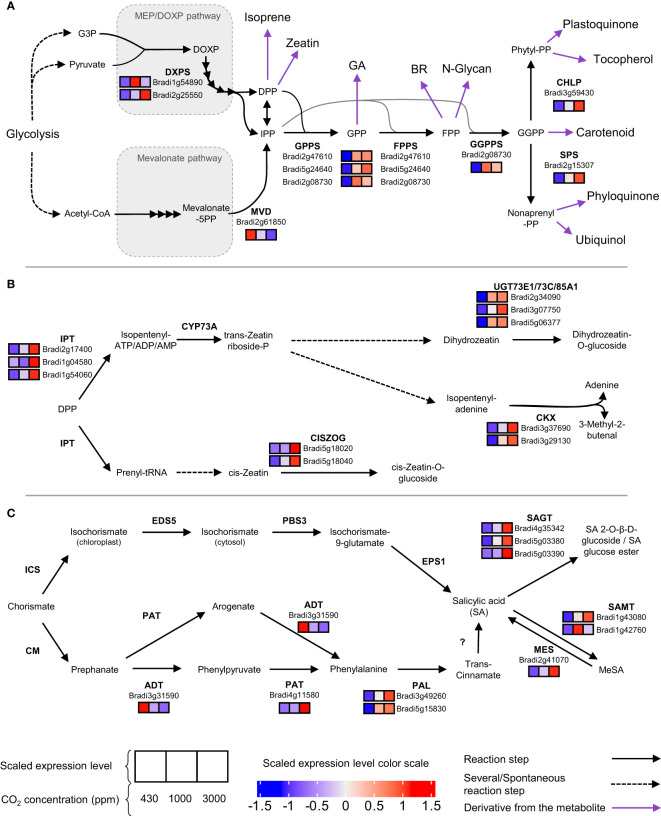
Terpenoid **(A)**, zeatin **(B)** and salicylic acid **(C)** pathways are upregulated at high CO_2_. For each chemical reaction, the substrate and product are separated by an arrow indicating its way. The enzyme catalyzing the reaction is written in bold on the side of the arrow. Under the enzyme names are written the gene accessions for the enzymes and shown as differentially expressed. Their normalised expression is depicted in three squares (from left to right: 430, 1000 and 3000 ppm) with the colour shade corresponding to the scaled expression level. Are only present expression value for differentially expressed genes. If the enzyme is unknown, a question mark is used. Purple arrows indicate a well-known derivative from the metabolite.

The enrichment analysis highlighted significant changes in the expression of genes involved in secondary metabolism (**Figure 5**). Cluster 3 showed enrichment for the “terpenoid backbone synthesis” term. Indeed, genes allowing the first step of the MEP pathway leading to terpenoid precursor synthesis were up-regulated at hCO_2_ (**Figure 6A**). Several downstream genes involved in the terpenoid backbone synthesis were also up-regulated. The terpenoid pathway allows the biosynthesis of diverse metabolites, among which zeatin, a cytokinin (CK) for which cluster 6 showed an enrichment (**Figure 5**). In addition, genes encoding enzymes allowing trans-zeatin (tZ) biosynthesis were up-regulated. The catabolism of cis-Zeatin (cZ) and tZ was also up-regulated (**Figure 6B**; **Table S4**). The enrichment analysis for the “terpene synthase activity” term confirmed this modulation of terpene metabolism, with the DEGs responsible for this enrichment being involved in the biosynthesis of terpene derivatives (**Table S3**).

Another major secondary metabolism pathway up-regulated by hCO_2_ corresponded to phenylpropanoids and derivatives, as highlighted by corresponding enrichment in clusters 6 and 9 (**Figure 5**). These genes allow the biosynthesis of flavanones, flavanols, and lignin. As well as Phe, two other aromatic amino acids (Tyr and Trp) may be important intermediates in the production of secondary metabolites. Although no term related to tyrosine (Tyr) metabolism was enriched, eight DEGs were identified encoding enzymes allowing biosynthesis of Tyr derivatives. Among these, one tyrosinase-encoding gene, *Bradi3g15040*, is a member of cluster 2, which showed a decrease in gene expression at hCO_2_. The seven others were included in clusters with opposite expression trends, namely clusters 6, 8 & 9 (**Table S4**). Concerning Trp, no genes responsible for its biosynthesis downstream of chorismate were up-regulated. Nevertheless, a Trp decarboxylase gene (*Bradi2g02370*) was up-regulated to similar levels for 1000 and 3000 ppm CO_2_ compared to 430 ppm (**Table S4**).

#### 3.4.3 Immunity and related signalling

A summary of DEGs related to immunity is shown in **Table S4**, including their predicted orthologs in rice or Arabidopsis. The plant immune system firstly acts by recognising stimuli partly thanks to receptor-like kinases/receptor-like proteins (RLK/RLP). Potential orthologs of *FLS2* and *SOBIR1* (RLKs in Arabidopsis) are two members of cluster 6, in which gene expression increased at hCO_2_. Eight additional DEGs coding for immunity-related leucine-rich repeat (LRR)-RLKs are comprised in clusters 1, 7, 6, 8 and 9 (**Table S4**). A putative *BIR1* ortholog, a member of cluster 9, was also up-regulated (**Figure 5**; **Table S4**). Resistance (R) proteins (NBS-LRR) also allow recognition of pathogen attacks. Genes encoding such proteins are present in each cluster (**Table S4**).

Signal transduction components downstream of pathogen recognition, such as mitogen-activated protein kinase (MAPK) modules, also featured among the DEGs. Members of MAPK modules were enriched in clusters 5 and 6 (**Figure 5**). MAPK-encoding genes are also present in clusters 2, 4 and 8 (**Table S4**). Moreover, WRKY transcription factors (TFs) are represented in various clusters (1, 4, 5, 6, 7 and 9), implying a signalling modulation by CO_2_ concentration (**Table S4**). Concerning calcium signalling, a calmodulin (*Bradi1g21095*) showed a downregulation, while a calcium-activated channel (*Bradi4g43061*) was up-regulated (**Table S4**). Furthermore, the “calmodulin binding” GO Molecular function enrichment for cluster 4 highlights a down-regulation of five calmodulin binding protein-coding genes. Orthologs of *PAD4* and *CBP60B*, well-known actors in plant defence signalling, were also among the DEGs. *PAD4* was up-regulated (cluster 9), and *CBP60B* orthologs were down-regulated (cluster 4).

Signal transduction also involves signalling induced by changes in phytohormone content. The impact of CO_2_ concentration on cytokinin-related expression was noted above. In addition, a gene coding for a type A RR (*Bradi5g11350*), a cytokinin-signalling repressor, is a member of cluster 9, showing an increased expression level at hCO_2_ (**Table S4**). The up-regulation of the phenylpropanoid pathway mentioned above can be correlated with the increase in biosynthesis of SA (**Figure 6C**). *BdPAL2* and *BdPAL8* are members of clusters 9 and 8, respectively, comprising DEGs with a higher expression at hCO_2_ (**Table S4**). Genes coding for enzymes allowing SA modification were up-regulated: SAGT, SAMT and MES. As well as genes involved in determining SA contents, genes encoding SA signalling components were also affected by hCO_2_. Two orthologs of *BRN1* (*Bradi2g60300*, *Bradi2g27260*) were grouped in cluster 2 and thus down-regulated at hCO_2_. An *NPR4* ortholog (*Bradi2g54340*), which was found in cluster 4, was also down-regulated. On the other hand, the *GRX480* ortholog (*Bradi2g46093*) was up-regulated as a member of cluster 9.

Other immunity-related phytohormones seemed to be regulated by hCO_2_. These included JA biosynthesis genes: two 13-LOX (*Bradi3g39980* and *Bradi5g11590*) and an AOS (*Bradi1g69330*) were up-regulated. Nevertheless, no modulation of JA-related signalling genes was observed at hCO_2_.

Ethylene biosynthesis seemed to be down-regulated, as reflected by the decreased transcripts for two ACO orthologs (*Bradi3g57620* and *Bradi2g35860*) (**Table S4**). Four genes coding for putative ethylene sensors were up-regulated at 3000 ppm CO_2_ with various trends at 1000 ppm (*Bradi3g55730*, *Bradi2g35080*, *Bradi3g56550* and *Bradi5g00700*; **Table S4**). Two ERFs, Bradi2g17610 and Bradi2g52370, were up and down-regulated at 1000 ppm, respectively, and both up-regulated at 3000 ppm CO_2_. *Bradi1g36530*, coding for an ERF binding protein allowing their degradation, followed the same trends as *Bradi2g52370*. Furthermore, *Bradi1g21372*, coding for an “ethylene induced calmodulin binding protein”, was down-regulated at high CO_2_ compared to 430 ppm. Moreover, RGA2 ortholog (*Bradi4g07902*), a repressor of GA signalling and component of immunity in *A. thaliana*, was up-regulated in hCO_2_. These results depict an extensive modulation of phytohormone biosynthesis and signalling, which might impact plant immunity.

Downstream of signalling, components that act on pathogen development are key in determining resistance, and include proteins encoded pathogenesis-related (*PR*) genes. At hCO_2_, eight genes coding for *PR* proteins were significantly up-regulated (**Table S4**). The only exception is a WAK (*Bradi1g02210*), a member of cluster 5, which showed a down-regulation at 1000 ppm and an up-regulation at 3000 ppm. Furthermore, four dirigent proteins are clustered and showed an up-regulation of gene expression at hCO_2_ (**Table S4**).

#### 3.4.4 DEGs associated with redox homeostasis

Several DEGs encoded proteins with known or potential roles in redox homeostasis. A gene encoding a chloroplastic malate dehydrogenase (*Bradi3g37140*) was included in cluster 3. This enzyme allows subcellular redox exchange through malate/oxaloacetate shuttling, and is thus potentially important for signal transduction between energy-producing/consuming organelles under hCO_2_ (Foyer and Noctor, 2020). In addition, *Bradi1g76330*, annotated as the catalase 2 gene, belongs to cluster 6. Concerning the ascorbate antioxidant system, one gene encoding ascorbate peroxidase (*Bradi5g03640*) and one for monodehydroascorbate reductase (*Bradi3g17120*) were up-regulated (clusters 9 and 8, respectively). Another clue suggesting enhanced oxidative pressure at hCO_2_ is the upregulation of the alternative oxidase (AOX; *Bradi5g20540*), an electron acceptor of the mitochondrial electron transport chain that acts to counter ROS generation. However, it should be noted that a gene coding for a superoxide dismutase was downregulated at hCO_2_ (*Bradi3g43070*; cluster 2).

## 4 Discussion

### 4.1 hCO_2_ impacts *B. distachyon* development

This work showed, first, that Bd responses to hCO_2_ imply a modulation of plant development at the vegetative stage and, even more obviously, at the reproductive stage. Both hCO_2_ conditions stimulated aspects of Bd development, although in general 1000 ppm had more positive effects, in line with previous studies on Bd, wheat and rice showing that CO_2_ concentrations between 550 and 900 ppm stimulate plant development in most of cases (Jakobsen et al., 2016; Blandino et al., 2020; Tcherkez et al., 2020; Ben Mariem et al., 2021). Indeed, FACE experiments over several decades have shown an increase in crop yield across species, with an average of 14 % (Ainsworth and Long, 2021). One aim of the present study was to include a CO_2_ concentration that was near-saturating for photosynthesis. At the vegetative stage, while for many genes 3000 ppm produced the same effect as 1000 ppm, albeit stronger, this did not translate to effects on parameters associated with growth and development. It therefore seems that 1000 ppm CO_2_ is sufficient to produce the maximal stimulation of Bd growth, at least in our culture conditions, suggesting that improved shoot development is most clearly observed at intermediate CO_2_ concentrations. At the vegetative stage, the specific response to 3000 ppm CO_2_, including modified expression of RuBisCO and other genes in cluster 3, may be indicative of a CO_2_ acclimation process (**Figure 5**).

### 4.2 *B. distachyon* primary and ROS-related metabolism are modulated by hCO_2_

The transcriptomics analysis of Bd confirmed that, as in other plants studied (Tausz-Posch et al., 2020), genes associated with primary metabolism and photosynthesis are up-regulated by hCO_2_. This adjustment was highlighted by enrichment for primary metabolism-related terms, most of which were influenced in proportion to CO_2_ concentration (**Figure 5**). N assimilation-associated gene expression was also enhanced, further pointing to a stimulation of primary metabolism. In this regard, it should be noted that plants were grown under a non-limiting supply of essential nutrients. Changes in CO_2_ concentration might also modulate signalling pathways closely associated with primary metabolism. Indeed, we observed a decrease in transcripts encoding five T6P synthases, which might impact trehalose-6-phosphate (T6P) metabolism and thus signalling associated with development and stress (Ponnu et al., 2011).

Increased primary metabolism at hCO_2_ and a resulting enhancement of ROS production at certain sites seem to be a characteristic shared among C_3_ flowering plant species (Noctor and Mhamdi, 2017). Our data are consistent with this notion, since hCO_2_ up-regulated several genes coding for enzymes that allow the biosynthesis of antioxidant compounds or enzymes permitting ROS detoxification. Increased primary metabolism might induce higher mitochondrial electron transport, and indeed the upregulation of an AOX supports the notion of increased ROS production at this location. Additionally, specific elements of hydrogen peroxide-detoxifying systems were also up-regulated at hCO_2_. A lower expression at hCO_2_ of a superoxide dismutase gene that is predicted to be chloroplastic may be suggestive of a complex or site-specific response. In any case, it is possible that adjustments in redox state might be part of the link between CO_2_ concentration and modulation of Bd immunity.

### 4.3 *B. distachyon* immunity and its related signalling, including phytohormones, are influenced by hCO_2_

It is striking that numerous elements involved in plant immunity were increased at hCO_2_, including RLKs, enzymes involved in phytoalexin biosynthesis, PR proteins, and modules such as MAPK modules and related TFs. Our targeted analysis of immunity components highlights the up-regulation in Bd of orthologs of several Arabidopsis genes that are established players in cell death and growth-defence trade-offs during the response to biotic stress (e.g., *FLS2*, *SOBIR1*, *BIR1* and *PAD4*; Liu et al., 2016). Interestingly, the MAPKs *BdMPK20-2* and *4*, whose orthologs are implicated in response to biotic stress, showed opposite trends at hCO_2,_ a phenomenon also observed for BdMKK4 and BdMKK10-2. While both these genes are up-regulated after MeJA and H_2_O_2_ treatments, only *BdMKK4*, which is up-regulated at hCO_2,_ is up-regulated after a CK treatment (Chen et al., 2012). Importance of CKs in the Bd CO_2_ response is discussed further below. Concerning WRKY, the gene family members up-regulated at hCO_2_ are also induced following infection with *F. graminearum* and *M. oryzae*, except for *BdWRKY51*, which is down-regulated during infection (Wen et al., 2014). All the WRKY orthologs down-regulated at hCO_2_ are also down-regulated during these biotic stresses (**Table S4**).

Components of innate immunity, such as transcripts encoding enzymes involved in monolignol production, were also increased at hCO_2_, as were genes encoding dirigent proteins involved in cell wall modifications (Paniagua et al., 2017). Cell wall modification and strengthening are important responses that reinforce physico-chemical barriers to resist pathogen entry and proliferation. This upregulation might be related to the higher plant shoot length measured under hCO_2,_ as cell wall reinforcement is required to support upright growth.

The SA concentration in Bd leaves was higher at 3000 ppm CO_2_ than 430 ppm CO_2_ (**Figure 2**). This result agrees with previous work showing that various species, including wheat, have a higher content in SA at hCO_2_ (Mhamdi and Noctor, 2016). However, in this previous study, the increase was shown at 1000 ppm CO_2_, a concentration that does not have a significantly different SA content in our study. Furthermore, the SA amount in the control condition is much higher than that of *H. vulgare*, *T. aestivum* or even *A. thaliana*. Concerning the basal SA content, values in Bd seem to be closer to *O. sativa*, which also exhibits high basal SA levels (Silverman et al., 1995). No variation in SA content was observed in response to inoculation with *M. grisea*, a hemibiotroph (Silverman et al., 1995), but this compound did increase in Bd seedlings following inoculation with *F. pseudograminearum* (Powell et al., 2017). The above points underline the species-specificity of both basal SA contents and their response to infection.

Alongside the general tendency to increased SA genes at hCO_2_ in Bd, genes encoding SA-modifying enzymes were also up-regulated, emphasising the impact of increased CO_2_ supply on the SA gene network. Bd genes annotated to encode SAMT and SAGT were grouped in clusters up-regulated at hCO_2_ (clusters 3, 6 and 9). Upregulation of SAMT suggests that the increase of SA might be related to SAR involving methyl-SA (Lefevere et al., 2020). The transcriptomic analysis also revealed a correlation between the increase in SA amount and an induction of pathways possibly responsible for SA biosynthesis in Bd (**Figure 6**). Two SA biosynthesis pathways have been described in plants, both of which depend on the shikimate pathway. One route depends on phenylalanine ammonia-lyase (PAL) and one involves isochorismate synthase (ICS) (Lefevere et al., 2020). Our data suggest that SA accumulation induced by high CO_2_ in Bd might involve the PAL pathway since two *PAL* genes were up-regulated under hCO_2_, whereas the single putative Bd *ICS* gene (*Bradi4g28670*) was not differentially expressed.

As mentioned above, hCO_2_ modulated the expression of genes related to ROS homeostasis. It is well-known that SA signalling is impacted by the cell redox state (Pieterse et al., 2012). Moreover, SA by itself can act on ROS homeostasis, for example, by inhibiting catalase activity (Conrath et al., 1995). Finally, the increased amounts of SA at 3,000 ppm could be related to our observations of growth decrease at the reproductive stage at this CO_2_ concentration. Indeed, it has been reported that the *A. thaliana sid2* mutant, with much reduced SA levels, shows greater growth under hCO_2_ than the wild-type, thereby pointing to a negative correlation between SA and growth at hCO_2_ (Mhamdi and Noctor, 2016).

Our analysis of gene enrichment reveals that hormones other than SA may also play an important part in the Bd CO_2_ response. The observed induction of predicted zeatin biosynthetic genes could indicate that hCO_2_ induces biosynthesis of CK alongside SA. Indeed, the biosynthesis of zeatin is induced by photosynthesis-generated sugars and is necessary for enhanced growth at hCO_2_ in *A. thaliana* (Kiba et al., 2019). This phytohormone is also involved in systemic nitrogen signalling, an important nutrient at hCO_2,_ since the C:N has been reported to be altered (Poitout et al., 2018; Tausz-Posch et al., 2020). However, these mechanisms involve a synthesis in the root. As our RNA-seq samples were taken from the shoots, they indicate that induction of CK synthesis is not restricted to root tissues.

As well as SA, hCO_2_ induced other genes potentially involved in the synthesis of antimicrobial compounds, such as aromatic amino acids and terpenoids (Balmer et al., 2013). For instance, serotonin, a Trp derivative, has been shown to have antimicrobial activity against *F. graminearum* (Pasquet et al., 2014). The Trp decarboxylase gene up-regulated at hCO_2_ is annotated to be involved in tryptamine and serotonin biosynthesis (**Table S4**). Concerning Tyr derivatives, genes responsible for biosynthesis of dhurrin, which is a cyanogenic glycoside implicated in plant defence (Gleadow and Møller, 2014), were also up-regulated under hCO_2_. Furthermore, a change in the contents of other amino acid can impact immunity (Liu et al., 2010; Stuttmann et al., 2011). It has been shown that isoprene and terpene derivatives have phytoalexin activity in both *A. thaliana* and monocots (Alméras et al., 2003; Schmelz et al., 2014; Frank et al., 2021). The impact of hCO_2_ on terpenoid-related gene expression in Bd is in line with previous reports on the relationship between isoprene biosynthesis, hCO_2_, and stress response (Lantz et al., 2019). However, a complex pattern was observed for these pathways in Bd. Notably, the “terpenoids” term is found in cluster 3, for which the expression of DEGs decreases at 3000 ppm CO_2_ compared to 1,000 ppm CO_2_. This difference should be noted since it might impact immunity.

Although caution must be exercised when comparing studies on different species, or using different experimental procedures (e.g., FACE vs. controlled environment), it is worth noting that in durum wheat (*T. turgidum* cv Regallo), exposure in the field to 700 ppm also increased levels of phenylpropanoid and terpene-related gene expression (Vicente et al., 2019). Nevertheless, our results contrast with another study in *T. aestivum* cv Norstar at the vegetative stage, which reported a decrease in biotic stress-related transcripts after growth at 700 ppm, notably two genes encoding PAL (Kane et al., 2013). In barley, a decrease in powdery mildew penetration at 700 ppm CO_2_ and an accumulation of phenylpropanoid pathway derivatives were observed whereas phenolamides, aromatic amino acid derivatives, were decreased after growth at hCO_2_ (Mikkelsen et al., 2015).

### 4.4 Perturbation resulting from growth at hCO_2_ induces increased susceptibility of *B. distachyon* to *M. oryzae*

Tests using *M. oryzae* showed that symptoms were more important after growth at hCO_2_ (**Figure 2**). However, no significant difference in fungal biomass in infected leaves was observed (**Figure S2**). A possible explanation for this result is that components of plant immunity are altered at hCO_2,_ leading to increased susceptibility to the fungal pathogen. Among them, a low to medium content in cytokinin was shown to induce a “cytokinin-induced susceptibility” (Chanclud et al., 2016; Albrecht and Argueso, 2017). Our transcriptome data have indicated a potential induction of zeatin biosynthesis by hCO_2,_ which could favour lesion development on Bd leaves following *M. oryzae* infection. In addition, the increased SA amount at hCO_2_ might not impact *M. oryzae* after its biotrophic stage but could rather impact the plant itself. It should be noted that CK can impact immunity by positively acting on SA signalling (Choi et al., 2010). However, although SA increases after growth at hCO_2_, a possible synergistic signalling with CK appears contradictory with the increase of Bd susceptibility to *M. oryzae*. Indeed, the resulting signalling should impact the biotrophic stage of this hemibiotrophic pathogen. Growth at hCO_2_ could result in more important symptoms due to changes on the host plant side.

Our observations for the Bd-*M. oryzae* interaction are in line with several studies that have reported of enhanced susceptibility of wheat to diverse pathogens after growth in this condition. For instance, in *T. aestivum* cv *Remus*, growth at 780 ppm CO_2_ increased disease symptoms to *Fusarium* head blight and *Septoria tritici* blotch (Váry et al., 2015). The effect was less significant for cultivars with higher resistance to the pathogens tested (Bencze et al., 2013; Váry et al., 2015; Cuperlovic-Culf et al., 2018). Concerning rice blast disease symptoms intensity, two FACE experiments on rice observed either an increase in symptom severity (Gória et al., 2013) or in disease incidence (Kobayashi et al., 2006). Although further studies are needed to decipher which components of immunity are responsible for the observed increase in susceptibility, our work has provided further evidence of links between hCO_2_ and biotic stress responses in plants, paving the way for studies to establish the roles of components involved in Bd immunity within the context of climate change.

## Data availability statement

The datasets presented in this study can be found in online repositories. The names of the repository/repositories and accession number(s) can be found below: https://www.ncbi.nlm.nih.gov/geo/,GSE229886.

## Author contributions

MD and GN conceived the project. LT, CM, JG, MG, AL-A, CP-LR, PR, and MD designed and conducted the experiments and analyses. LT, MD, and GN drafted the manuscript. All authors contributed to the article and approved the submitted version.
