# Supplementary material for: Impact of high atmospheric carbon dioxide on the biotic stress response of the model cereal species Brachypodium distachyon
Source: Front Plant Sci. 2023 Aug 16;14:1237054. doi: 10.3389/fpls.2023.1237054 (PMC10469009; doi:10.3389/fpls.2023.1237054)
Supplement: Supplementary file 1 [file Presentation_1.pptx]

## Slide 1
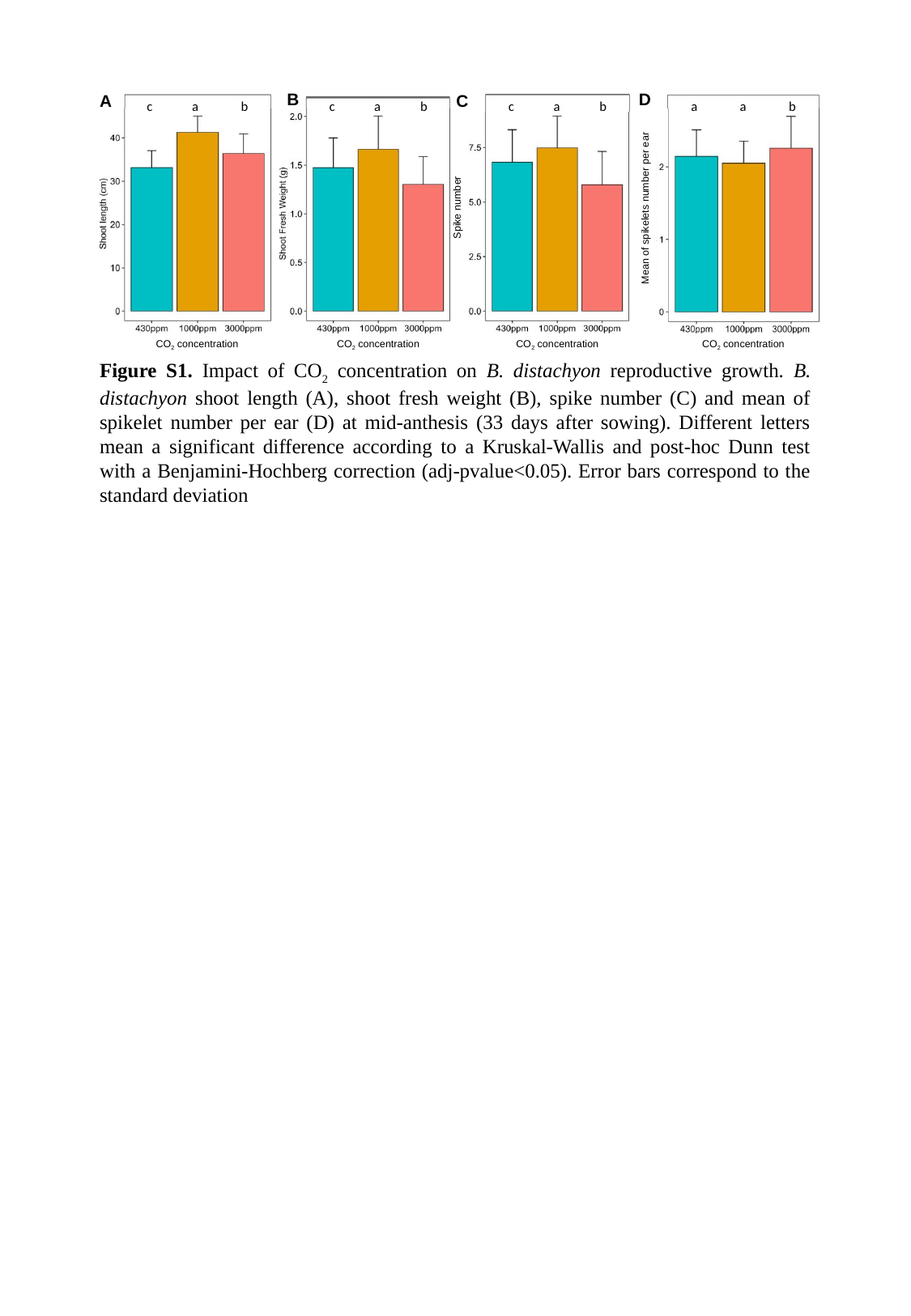

B
D
A
C
c a b
c a b
c a b
a a b
Spike number
Mean of spikelets number per ear
CO2 concentration
CO2 concentration
CO2 concentration
CO2 concentration
Figure S1. Impact of CO2 concentration on B. distachyon reproductive growth. B. distachyon shoot length (A), shoot fresh weight (B), spike number (C) and mean of spikelet number per ear (D) at mid-anthesis (33 days after sowing). Different letters mean a significant difference according to a Kruskal-Wallis and post-hoc Dunn test with a Benjamini-Hochberg correction (adj-pvalue<0.05). Error bars correspond to the standard deviation

## Slide 2
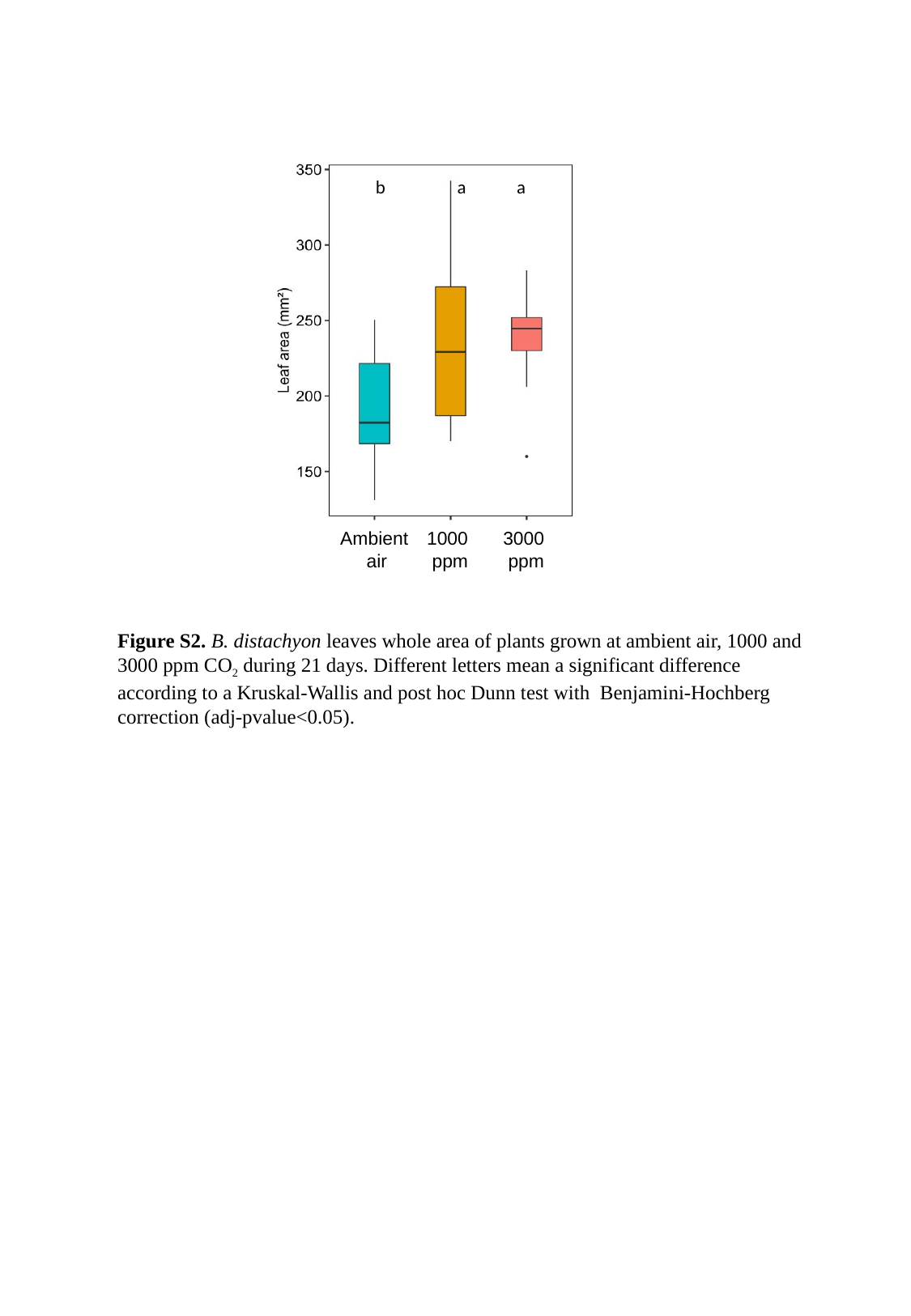

b a a
Ambient
 air
1000
ppm
3000
ppm
Figure S2. B. distachyon leaves whole area of plants grown at ambient air, 1000 and 3000 ppm CO2 during 21 days. Different letters mean a significant difference according to a Kruskal-Wallis and post hoc Dunn test with Benjamini-Hochberg correction (adj-pvalue<0.05).

## Slide 3
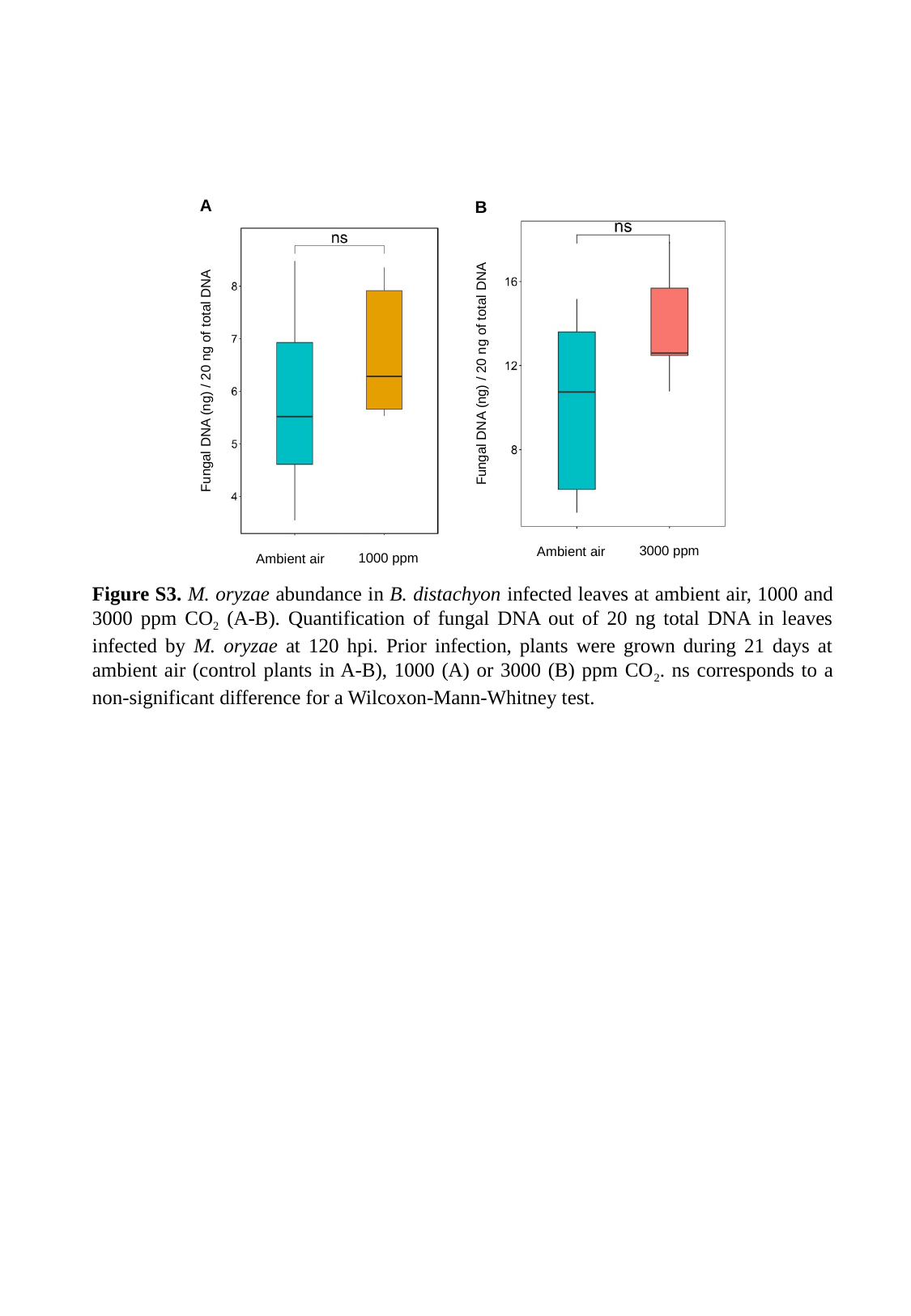

A
B
Fungal DNA (ng) / 20 ng of total DNA
Fungal DNA (ng) / 20 ng of total DNA
3000 ppm
Ambient air
1000 ppm
Ambient air
Figure S3. M. oryzae abundance in B. distachyon infected leaves at ambient air, 1000 and 3000 ppm CO2 (A-B). Quantification of fungal DNA out of 20 ng total DNA in leaves infected by M. oryzae at 120 hpi. Prior infection, plants were grown during 21 days at ambient air (control plants in A-B), 1000 (A) or 3000 (B) ppm CO2. ns corresponds to a non-significant difference for a Wilcoxon-Mann-Whitney test.

## Slide 4
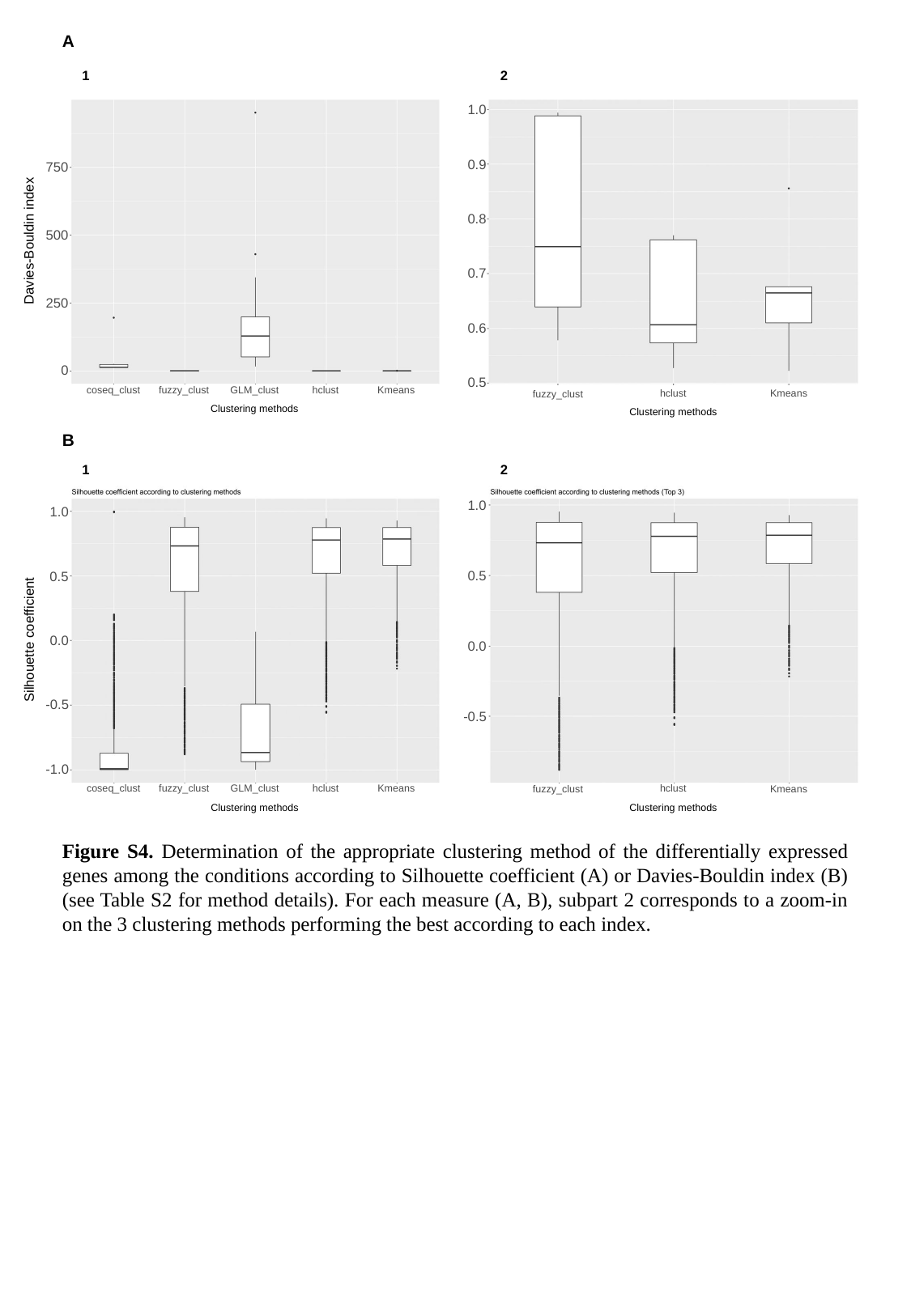

A
1
2
 1.0
 0.9
 0.8
 0.7
 0.6
 0.5
hclust
Kmeans
fuzzy_clust
Clustering methods
Davies-Bouldin index
750
500
250
 0
coseq_clust
fuzzy_clust
GLM_clust
hclust
Kmeans
Clustering methods
B
1
2
 1.0
 0.5
 0.0
-0.5
hclust
Kmeans
fuzzy_clust
Clustering methods
 1.0
Silhouette coefficient
 0.5
 0.0
-0.5
-1.0
coseq_clust
fuzzy_clust
GLM_clust
hclust
Kmeans
Clustering methods
Figure S4. Determination of the appropriate clustering method of the differentially expressed genes among the conditions according to Silhouette coefficient (A) or Davies-Bouldin index (B) (see Table S2 for method details). For each measure (A, B), subpart 2 corresponds to a zoom-in on the 3 clustering methods performing the best according to each index.

## Slide 5
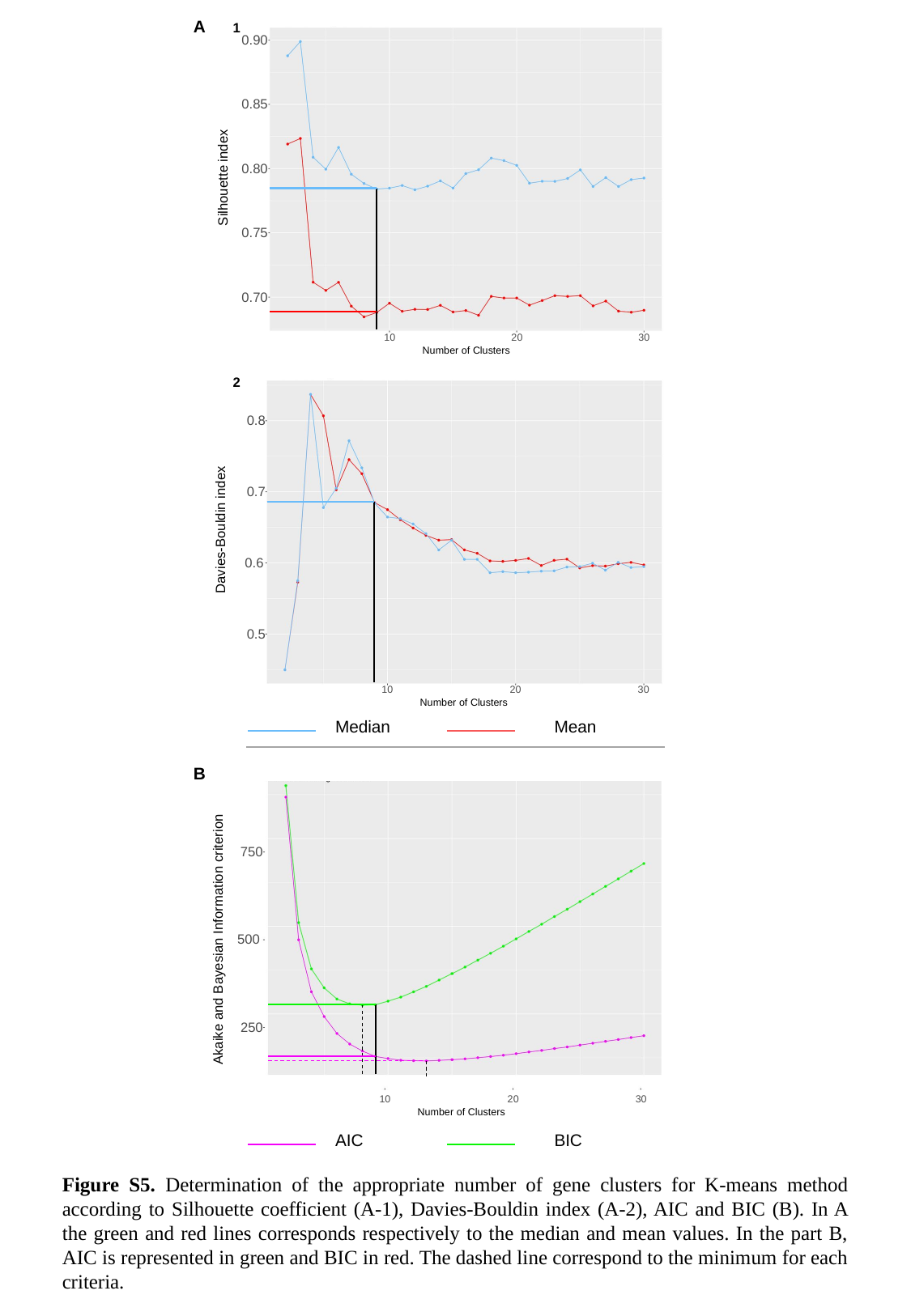

A
1
0.90
Silhouette index
0.85
0.80
0.75
0.70
30
20
10
Number of Clusters
2
Davies-Bouldin index
 0.8
 0.7
 0.6
 0.5
30
20
10
Number of Clusters
Median
Mean
B
Akaike and Bayesian Information criterion
 750
500
 250
30
20
10
Number of Clusters
AIC
BIC
Figure S5. Determination of the appropriate number of gene clusters for K-means method according to Silhouette coefficient (A-1), Davies-Bouldin index (A-2), AIC and BIC (B). In A the green and red lines corresponds respectively to the median and mean values. In the part B, AIC is represented in green and BIC in red. The dashed line correspond to the minimum for each criteria.

## Slide 6
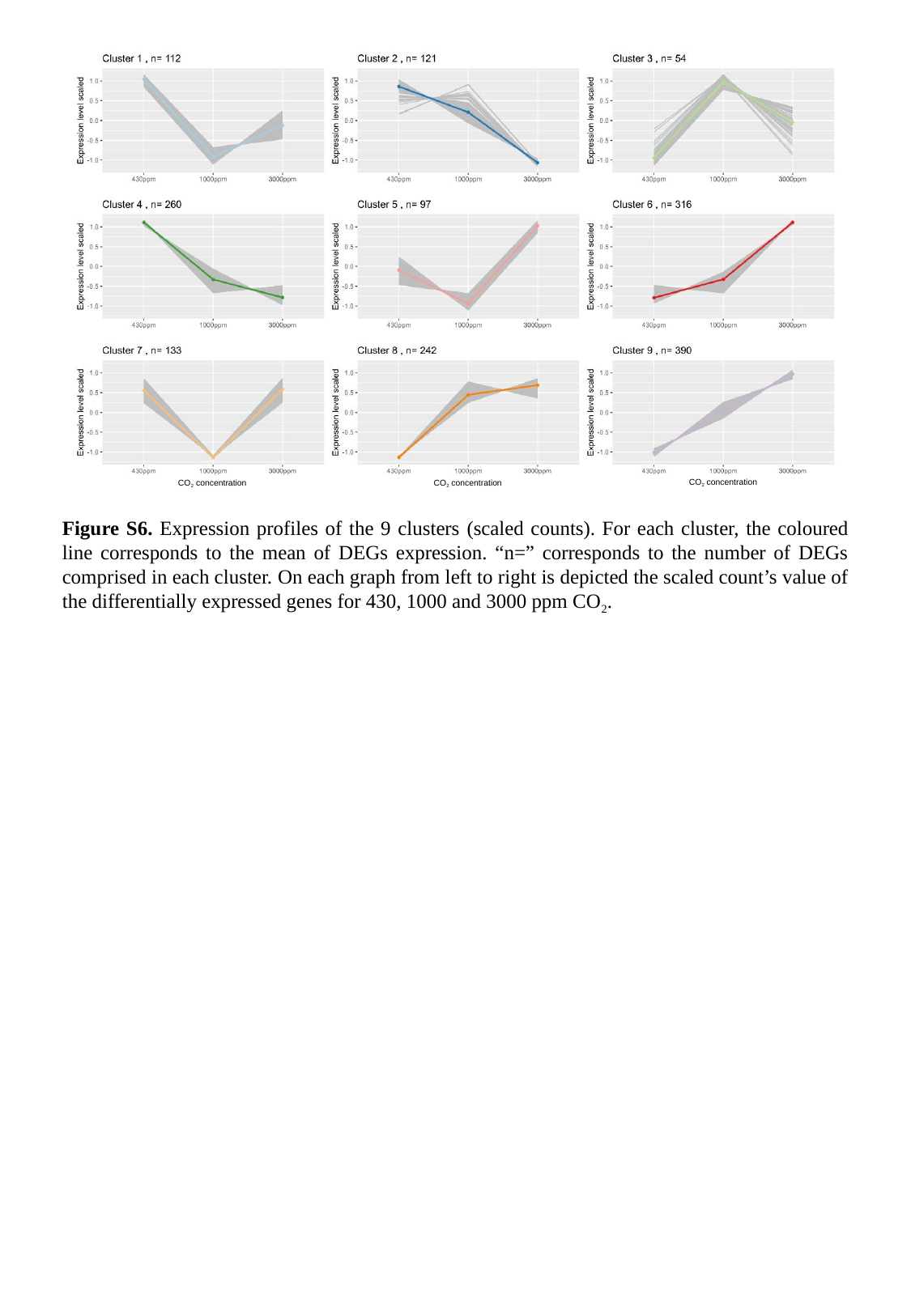

CO2 concentration
CO2 concentration
CO2 concentration
Figure S6. Expression profiles of the 9 clusters (scaled counts). For each cluster, the coloured line corresponds to the mean of DEGs expression. “n=” corresponds to the number of DEGs comprised in each cluster. On each graph from left to right is depicted the scaled count’s value of the differentially expressed genes for 430, 1000 and 3000 ppm CO2.
